# Supplementary material for: Thin films of the α-quartz SixGe1-xO2 solid solution
Source: Sci Rep. 2022 Feb 7;12:2010. doi: 10.1038/s41598-022-05595-z (PMC8821611; doi:10.1038/s41598-022-05595-z)
Supplement: Supplementary file 1 — Supplementary Information. [file 41598_2022_5595_MOESM1_ESM.pdf]

Thin films of the α-quartz $Si_xGe_{1-x}O_2$ solid-solution

Silang Zhou[1], Jordi Antoja-Lleonart[1], Václav Ocelík[1], Beatriz Noheda[1]*

1.Zernike Institute for Advanced Materials, University of Groningen, Nijenborgh 4, 9747AG Groningen, The Netherlands

Corresponding author: b.noheda@rug.nl

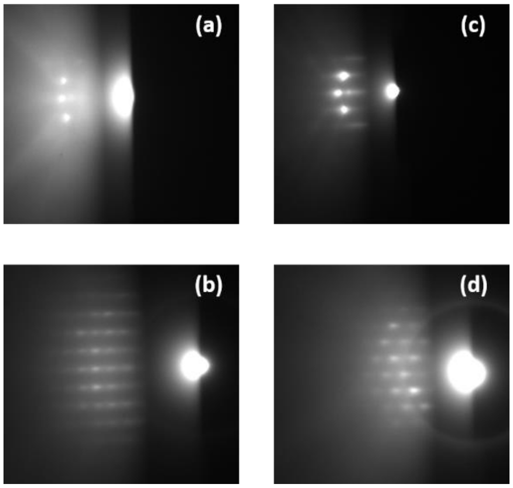

Fig. S 1. RHEED pattern evolution of epitaxial growth of GeO_2 on Z-cut quartz (a-b) and Y-cut quartz (c-d). (a) and (c) are the patterns of the substrate before the growth, and (b) and (d) are the patterns of the thin film during deposition. These films are deposited at 800 Celsius.

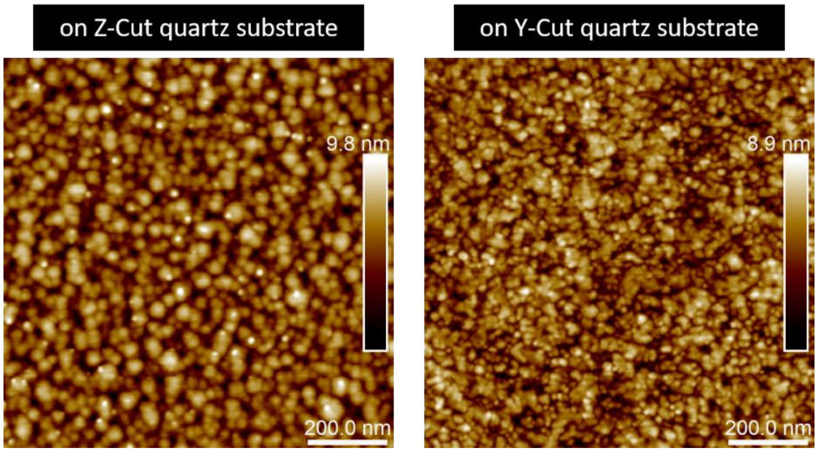

Fig. S 2. AFM images of epitaxial GeO_2 thin films on quartz substrates.

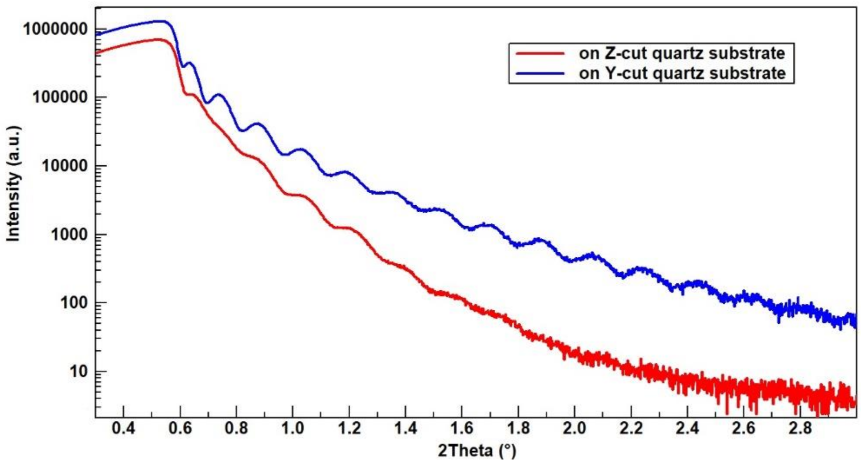

Fig. S 3. X-ray reflectivity of the epitaxial GeO_2 thin films on quartz substrates.

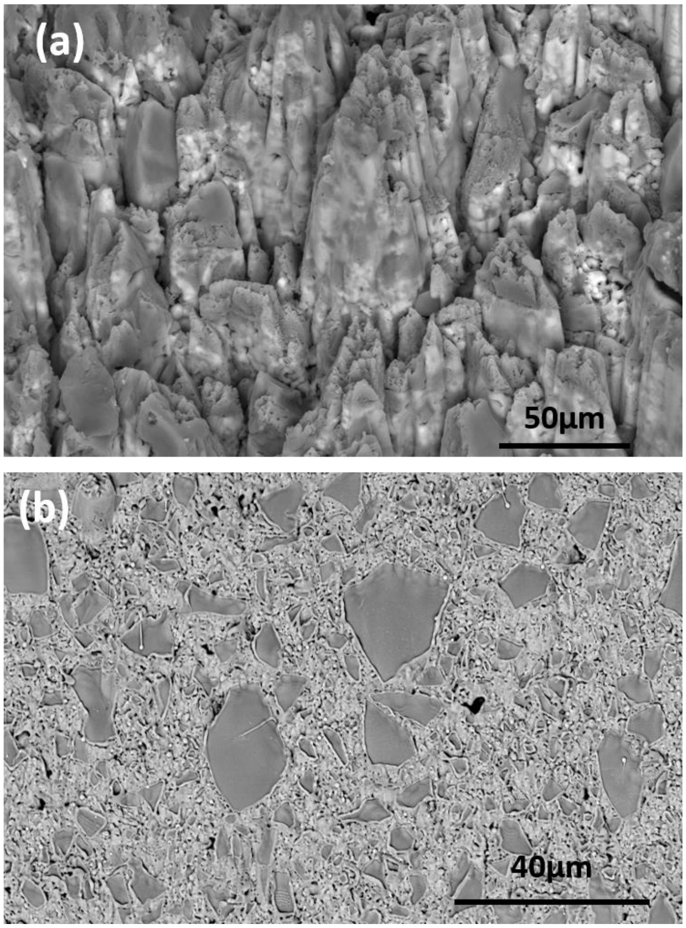

Fig. S 4.SEM images with CBS detector of targets after laser ablation. (a) target (7) $Si_{0.66}Ge_{0.33}Cs_{0.01}O_2$ which is composed of quartz (GeO_2) and quartz (SiO_2). After ablation, columnar features appear and the dark contrast at the top of the columns suggests it is covered by a layer of SiOx. (b) target $Si_{0.71}Ge_{0.19}O_2$ which is composed of quartz (GeO_2) and cristobalite (SiO_2). The morphology of this target is much flatter than in (a).

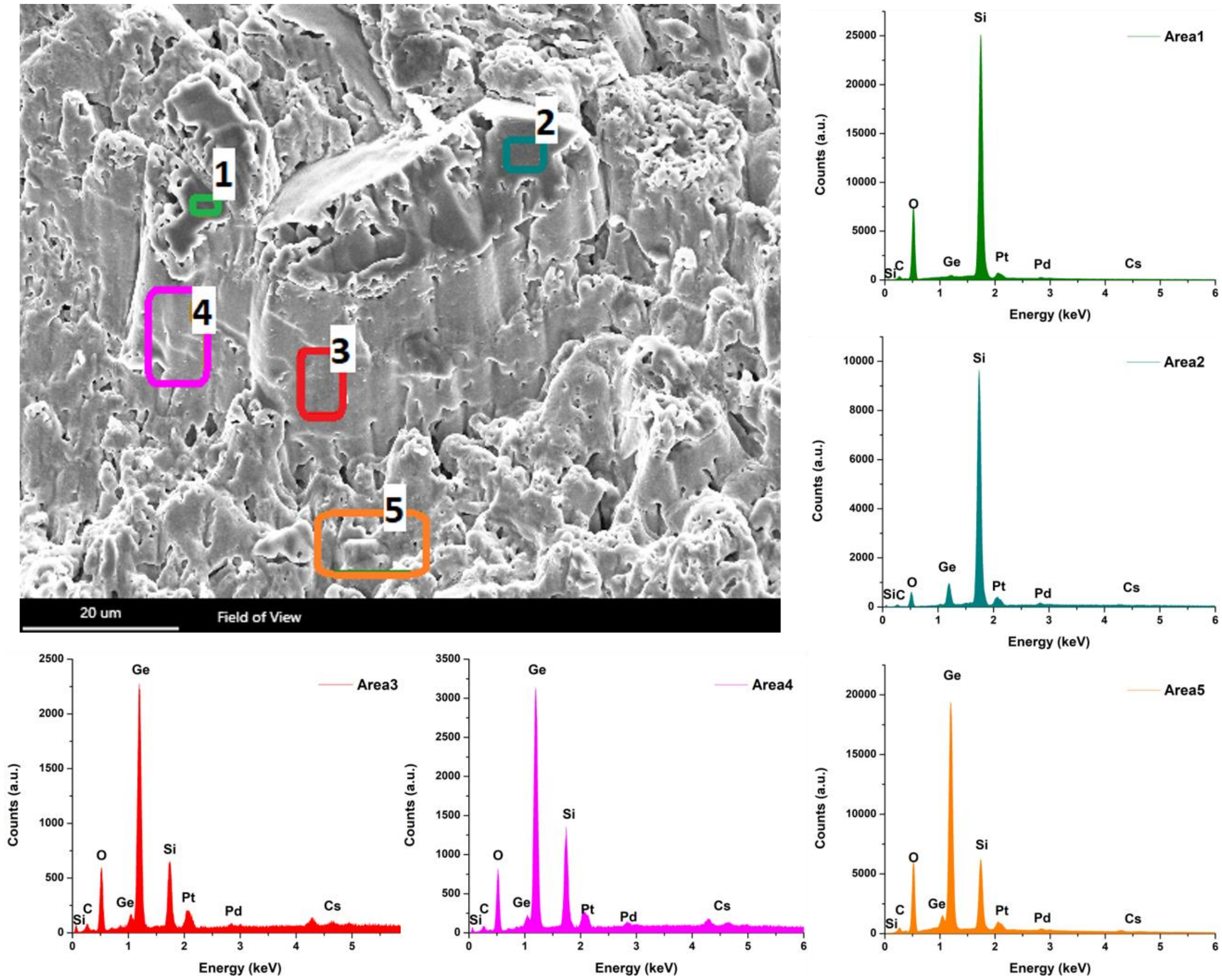

Fig. S 5.EDS analysis of the columnar morphology of target(7) $Si_{0.66}Ge_{0.33}Cs_{0.01}O_2$ after laser ablation, showing the top of the column is covered by SiOx or Si.

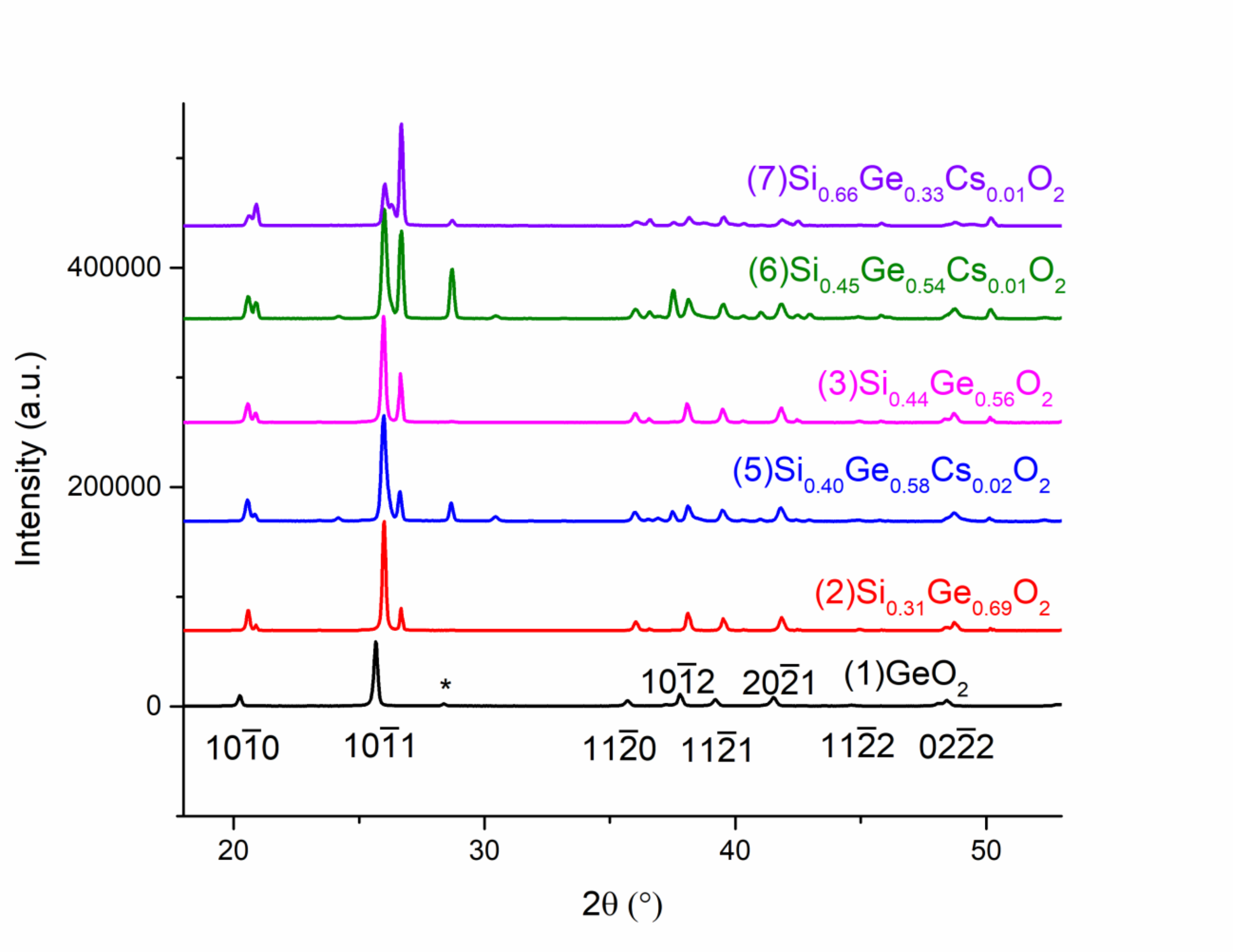

*Fig. S 6. XRD of the targets made from SiO_2 and GeO_2 powders shows the targets are composed of SiO_2 quartz and GeO_2 quartz crystals rather than solid solutions. The peak marked in * can be indexed with Germanium (111).*

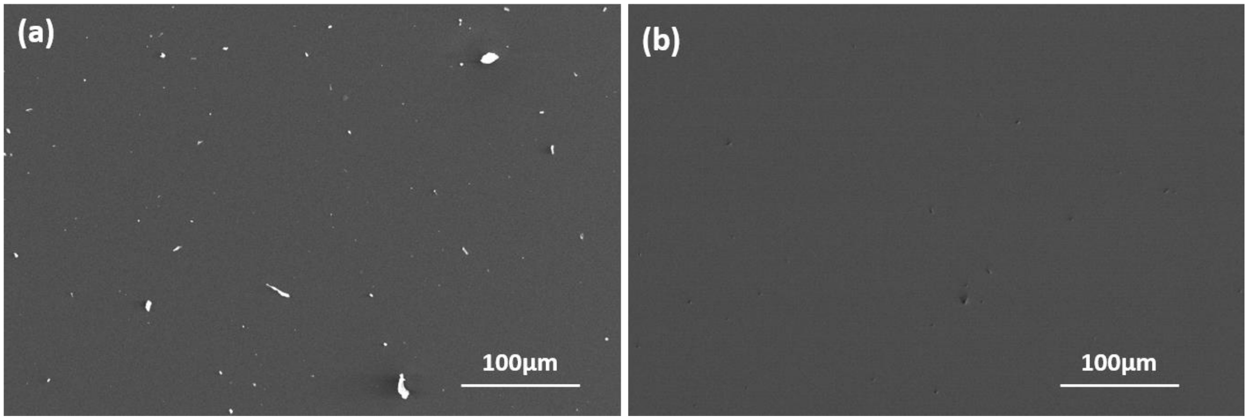

Fig. S 7. SEM images of thin films of (a) $Si_{0.48}Ge_{0..52}O_2$ deposited from target (7) $Si_{0.66}Ge_{0.33}Cs_{0.01}O_2$, where the SiO_2 is in quartz phase, and (b) $Si_{0.75}Ge_{0.25}O_2$ deposited from target (4) $Si_{0.71}Ge_{0.29}O_2$, where the SiO_2 is in cristobalite phase. The amount of particulates are greatly reduced in (b).

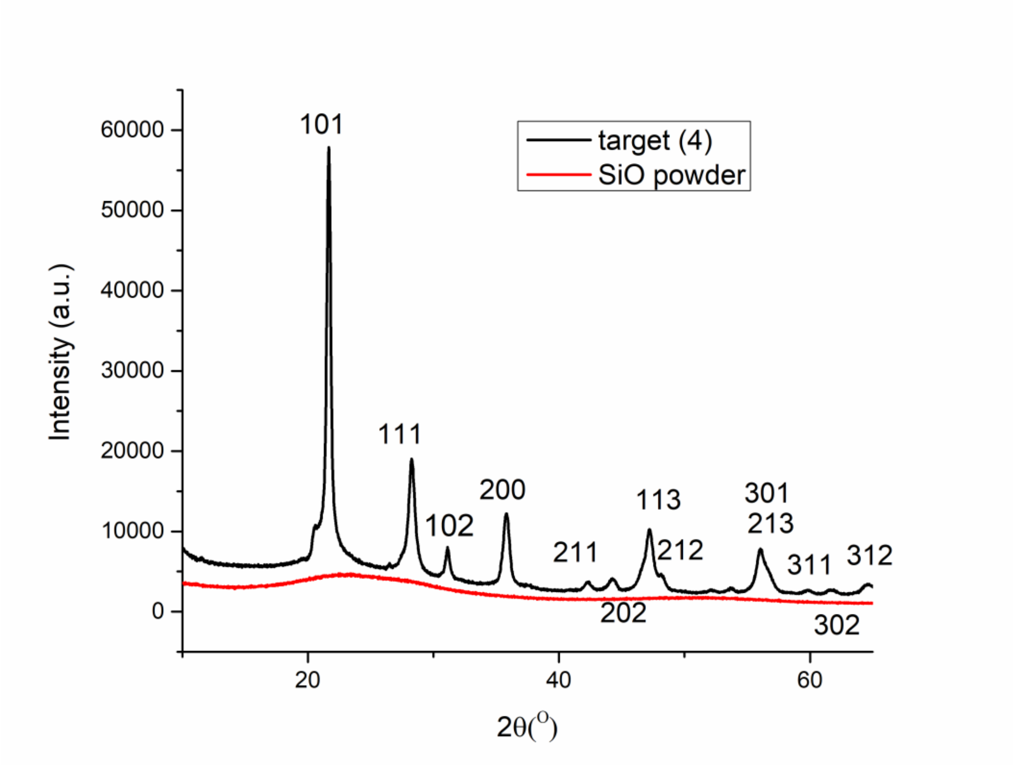

Fig. S 8. XRD patterns of target (4) $Si_{0.71}Ge_{0.19}O_2$ in Table. S1 which is consistant with the cristobalite phase of SiO_2, and the SiO powder which is amorphous.

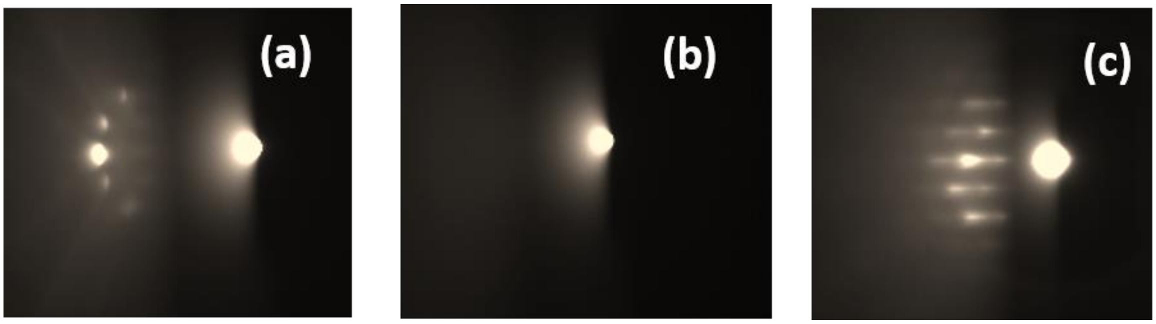

Fig. S 9. RHEED evolution of GeO_2 thin films on quartz substrates. These films are deposited at 600 Celsius and annealed at 800 Celsius.(a) before the growth, (b) during the growth, after several tens of pusles, the RHEED patter disappear (c) after the annealing the RHEED pattern recovers showing elongated rods.

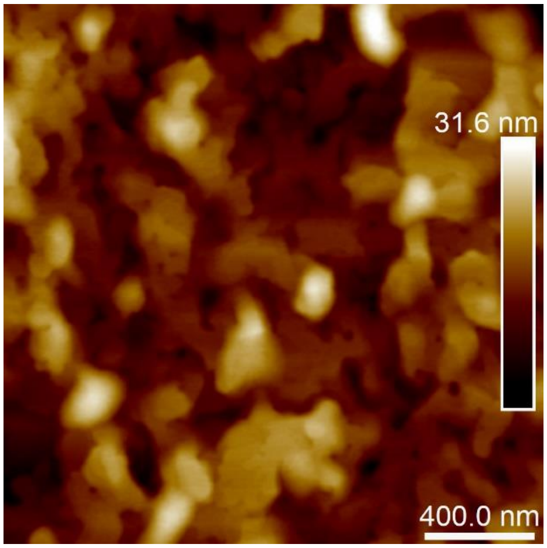

Fig. S 10. AFM scan of the flat area of $Si_{0.48}Ge_{0.52}O_2$ thin film on Y-cut quartz substrate.

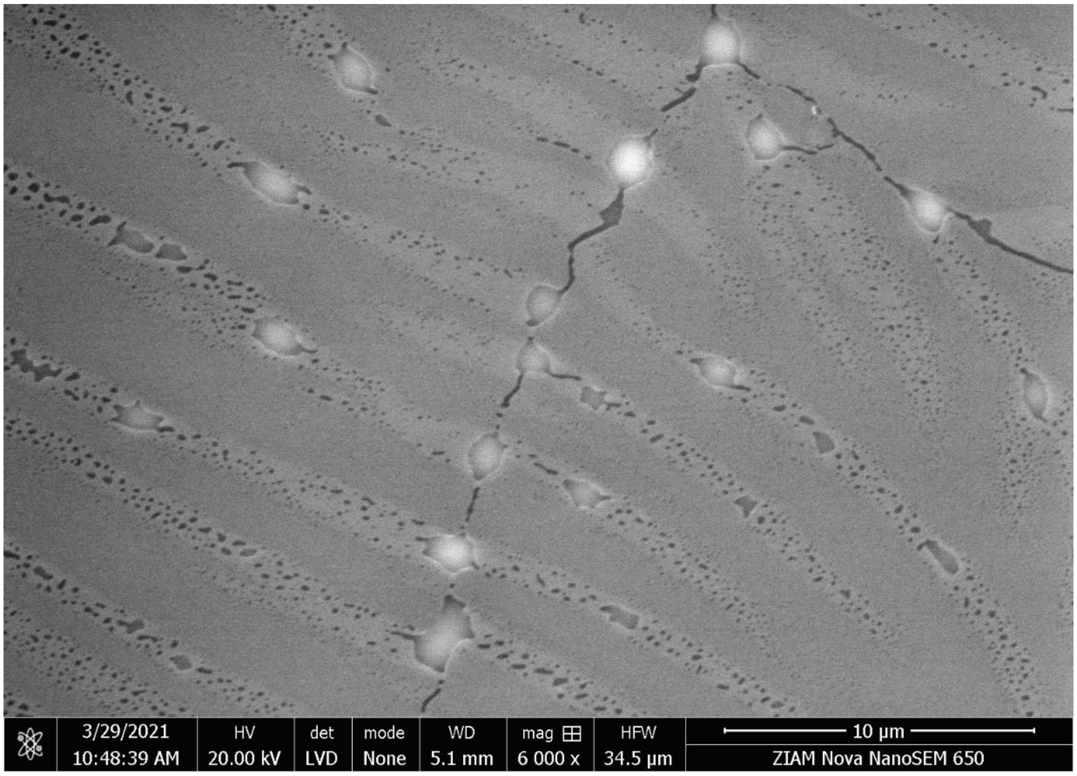

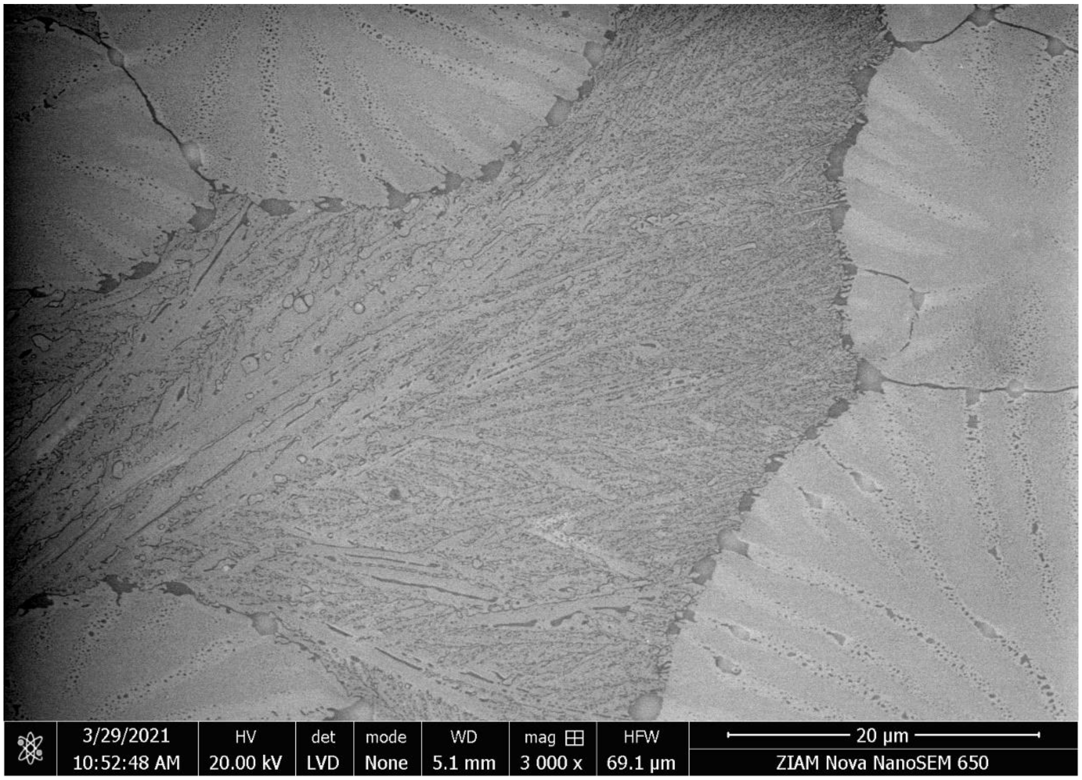

Fig. S 11. Top: SEM image of fibous structures of $Si_{0.75}Ge_{0.25}O_2$ *domains shows many small holes are inside the stripes and the stripes can connect to each other across the domains. Bottom: SEM image of low-crystallinity area in between the domains.*

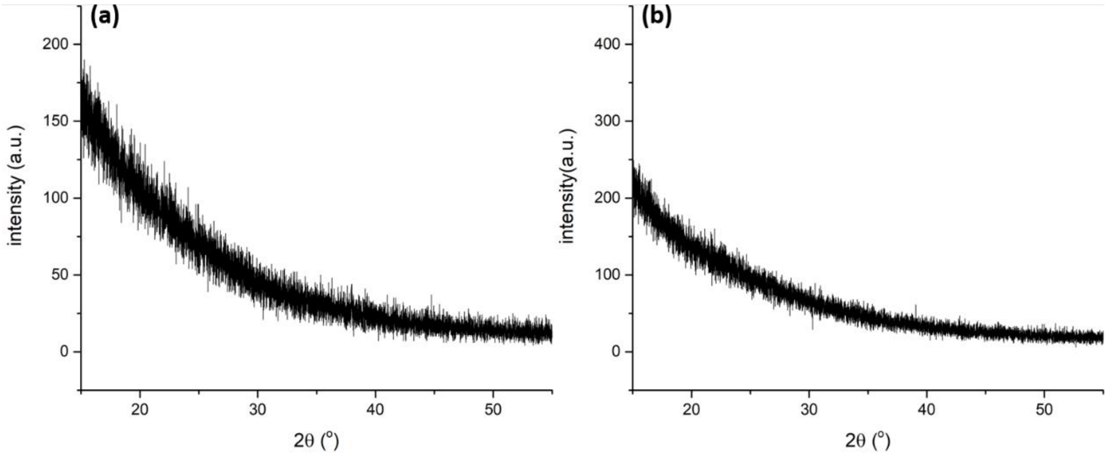

Fig. S 12. GIXRD of: (a) Thin film of $Si_{0.75}Ge_{0.25}O_2$ on quatz substrates. (b) thin film of $Si_{0.21}Ge_{0.79}O_2$ on sapphire substrates. Both diagaiams show no peaks because thin film in (a) is oriented while thin film in (b) is amorphous.

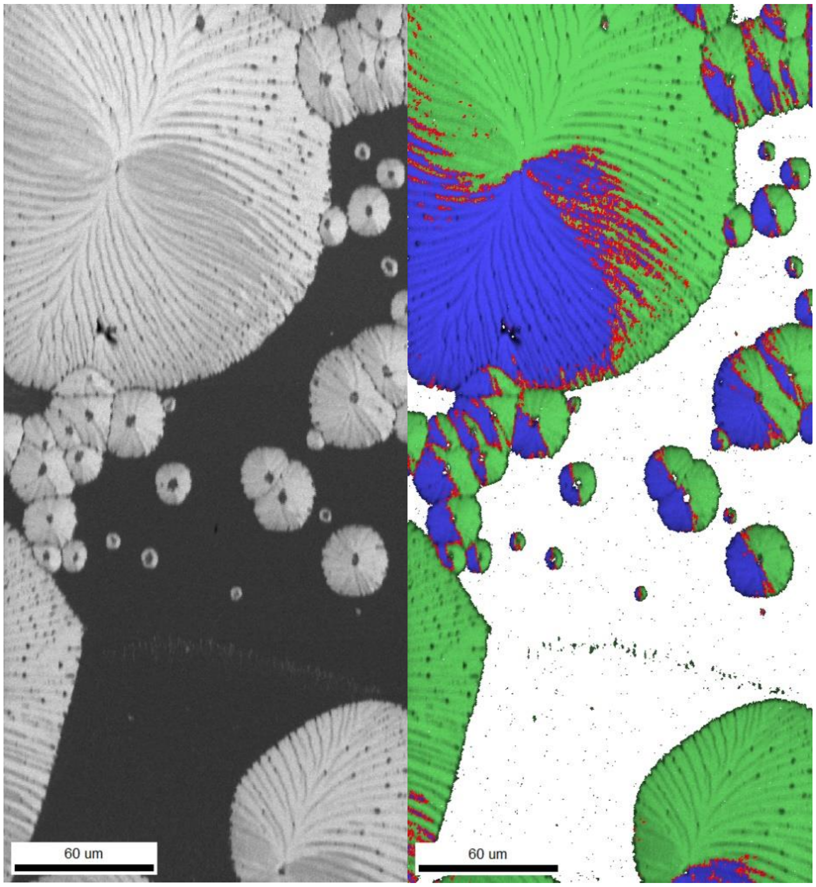

Fig. S 13. EBSD analysis of domains on $Si_{0.75}Ge_{0.25}O_2$ thin films. (left) Image Quality map where the greyscale represents the crystallinity. (right) Orientation map showing the same orientations as on Fig. 6 in the main text and the red lines are the Dauphiné twin boundaries. Notice the twin boundary evolves with the size of the domains. For smallest domains, the twin boundary is a nearly vertical straight lines. With the growth of the domains, the twin boundary rotate conterclockwise and finally forming a Yin Yang pattern in the big domains.

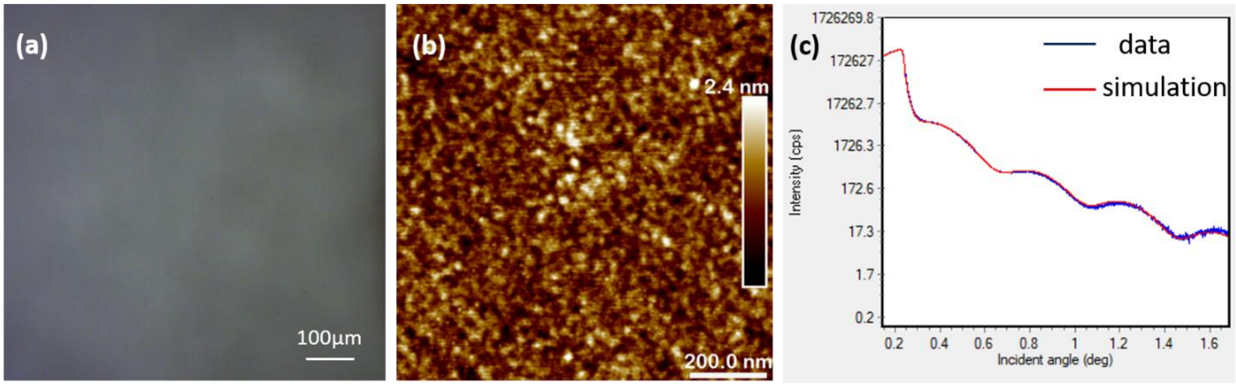

Fig. S 14. Thin film of SiO_2 on quartz substrate with a total number of pulses of 1200. (a) optical microscopy image shows there is no feature on the film. (b) AFM image os the thin surface, showing flat surface. (c) Reflectivity of the thin film with the simulation parameters: film thickness of 10.8nm, density of 2.24g/cm^3, and roughness of 0.48nm.

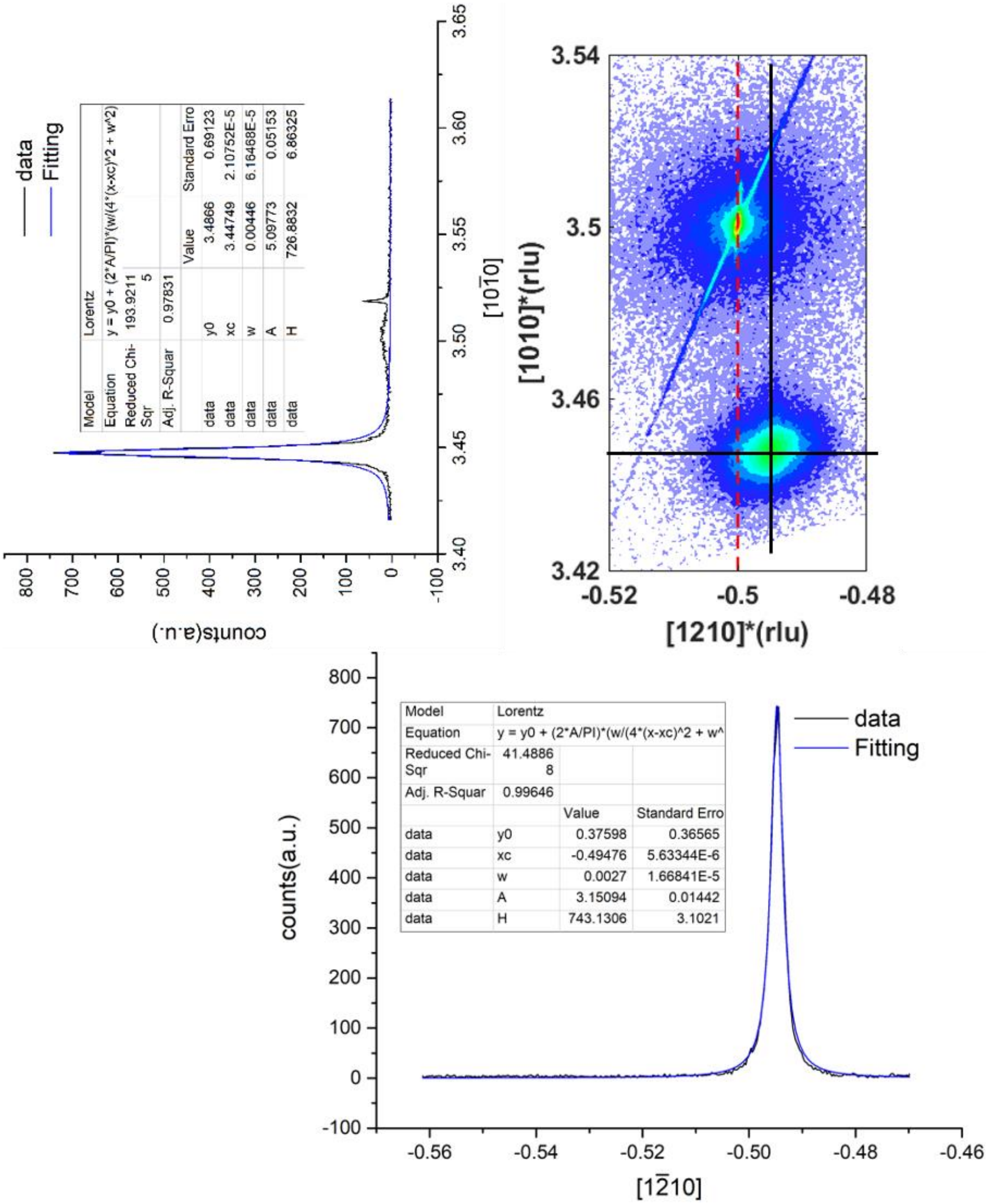

Fig. S 15. An example shows how the lattice parameters are extracted by Lorentzian fitting.

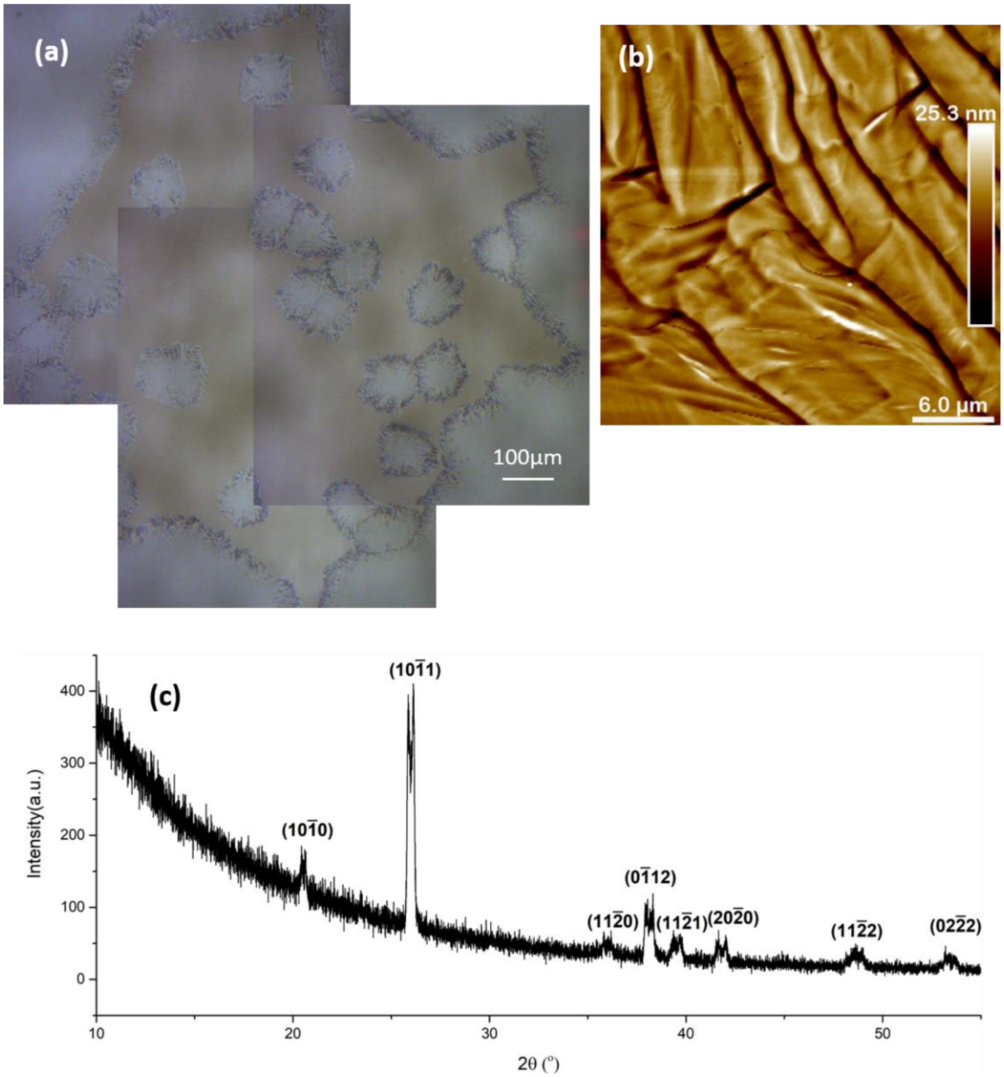

Fig. S 16. Thin film of GeO_2 on Al_2O_3 substrates annealed at 880 ℃. (a): optical microscopy images of the center of the thin film. The contunous crystallization starts from the edge of the sample and propagate towards the center of the film, and only the center is not fully crystallized. In the center of film, separate quartz crystals with diameter about 100 µm can be observed. (b): AFM image of the continuous crystallization part shows the wavy patten. (c): GIXRD shows the poly-crystallinity of the film.

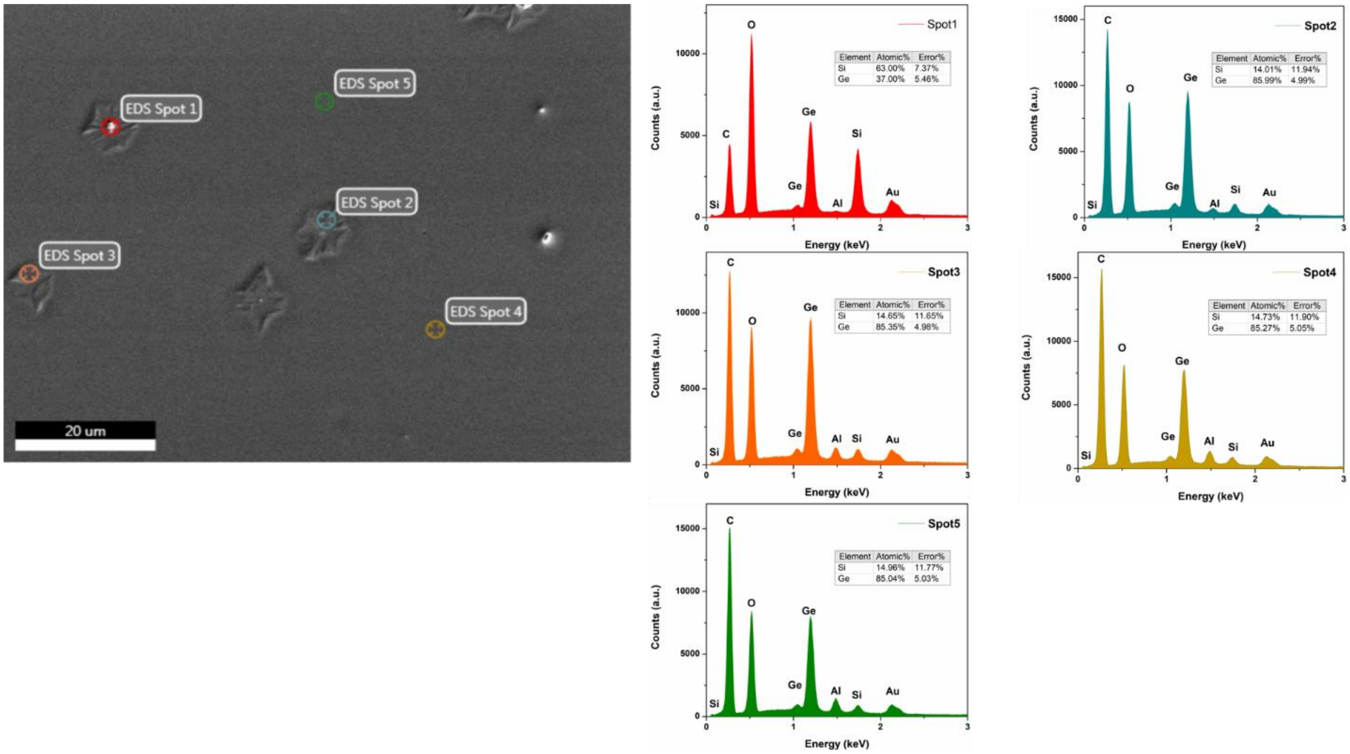

Fig. S 17. EDS point analysis of thin film of $Si_{016}Ge_{0.84}O_2$ on sapphire substrates showing except the spot1 which is on the core of the crystal, the film is homogenous in terms of composition.

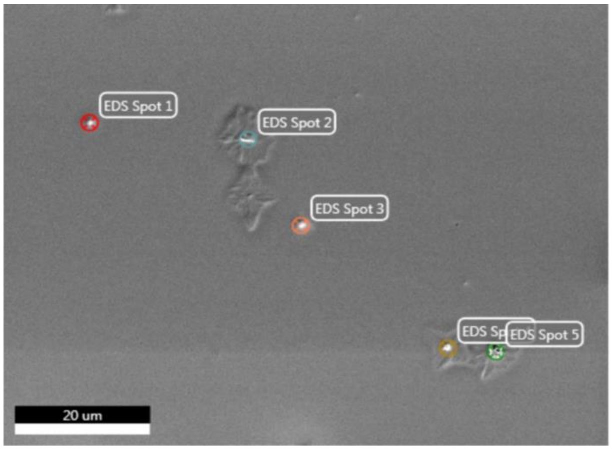

Spot	Si%	Ge%	Si Error%	Ge Error%
1	77.48	22.52	6.65	5.71
2	85.54	14.46	6.42	6.33
3	76.27	23.73	6.78	5.70
4	73.40	26.60	6.89	5.63
5	21.43	78.57	9.76	4.85

Fig. S 18. EDS point analysis on the cores of the crystals and the round bumps on the amorphous area, showing they have quite different composition comparing to the average of the thin film of $Si_{016}Ge_{0.84}O_2$.

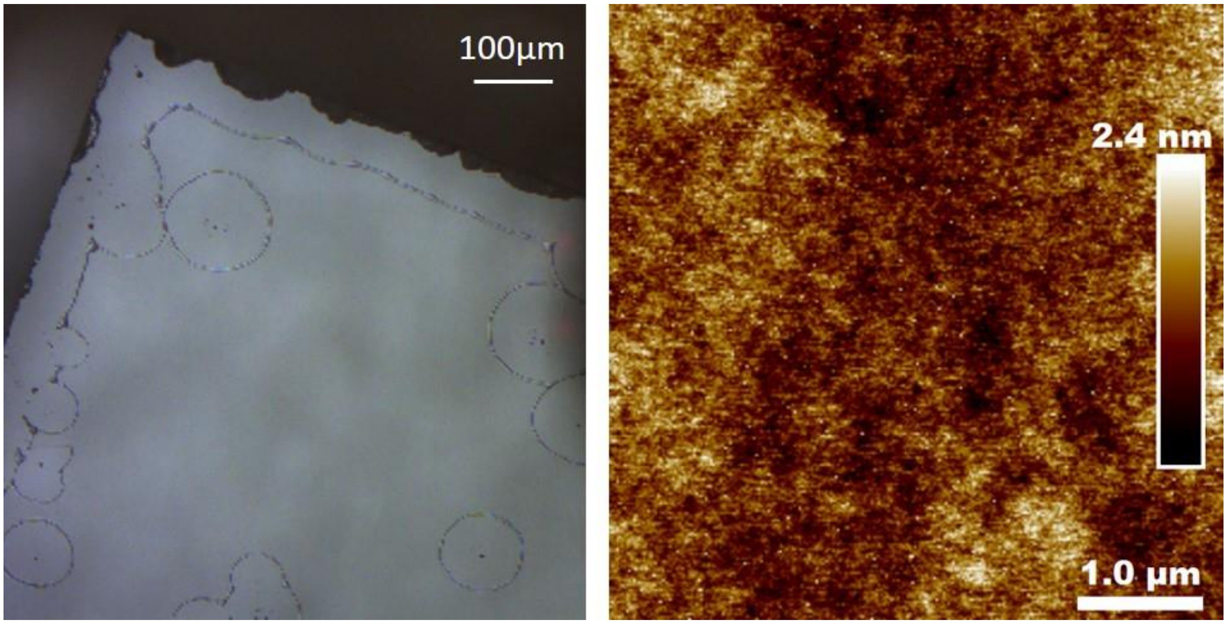

Fig. S 19. Thin film of GeO_2 on Al_2O_3 substrates deposited at 800℃. Left: Optical microscopy image shows the thin film starts evaporate from the edge and circular evaporation pits are also present. Right: AFM image of the thin film reveal flat surface without any features suggesting it is amorphous.

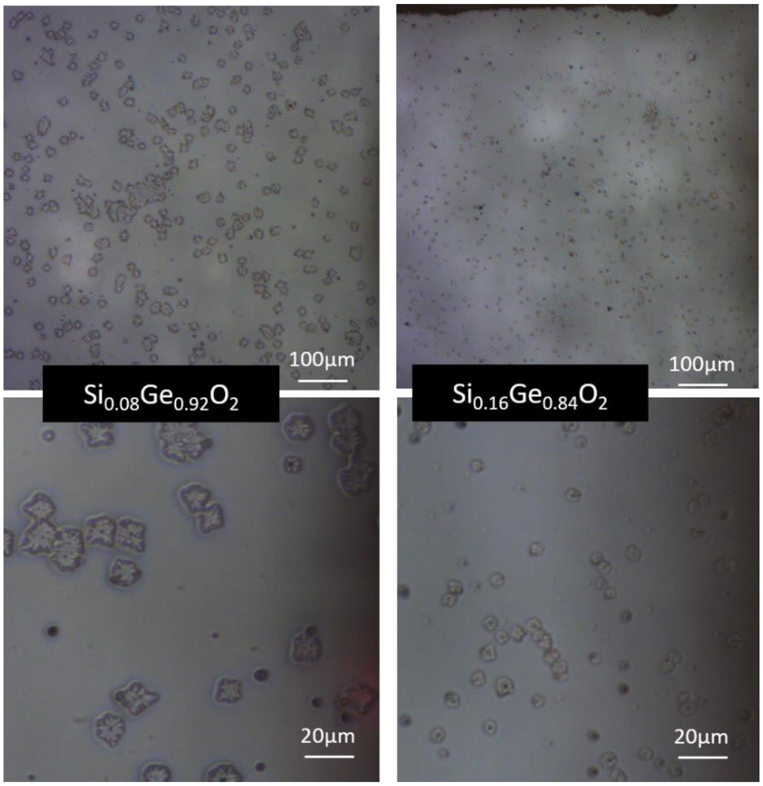

Fig. S 20. Optical microscopy images of thin films of $Si_{0.08}Ge_{0.92}O_2$ and $Si_{016}Ge_{0.84}O_2$ on sapphire substrates with different zoom in.

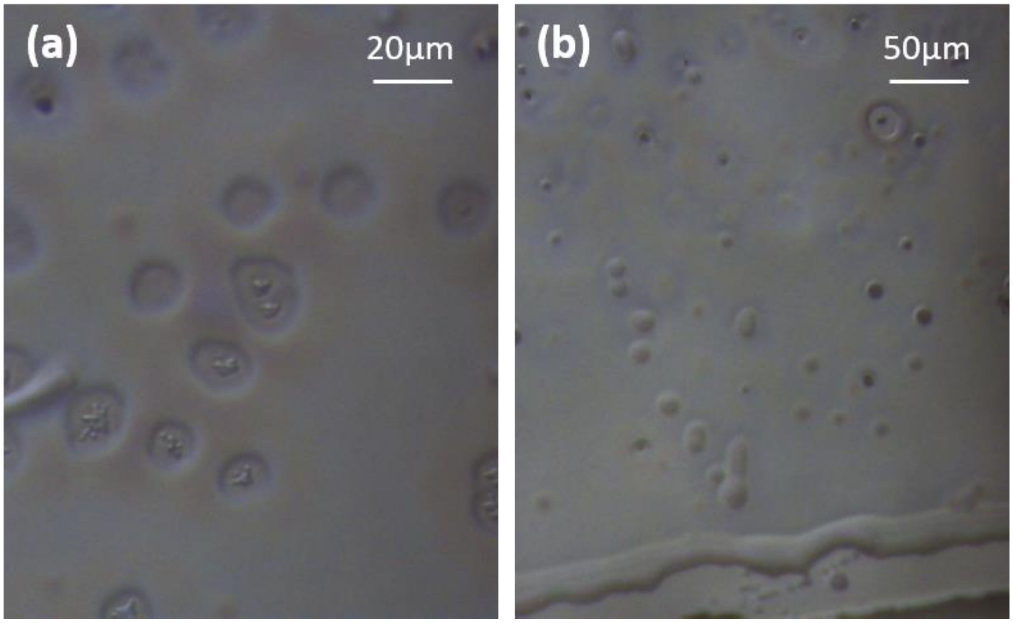

Fig. S 21. Thin film of $Si_{0.08}Ge_{0.92}O_2$ on sapphire substrate with longer annealing (2 hours) showing the quartz crystals start evaporates, forming evaporation pits. And the whole film starts evaporate from the edge as shown in the bottom in (b).

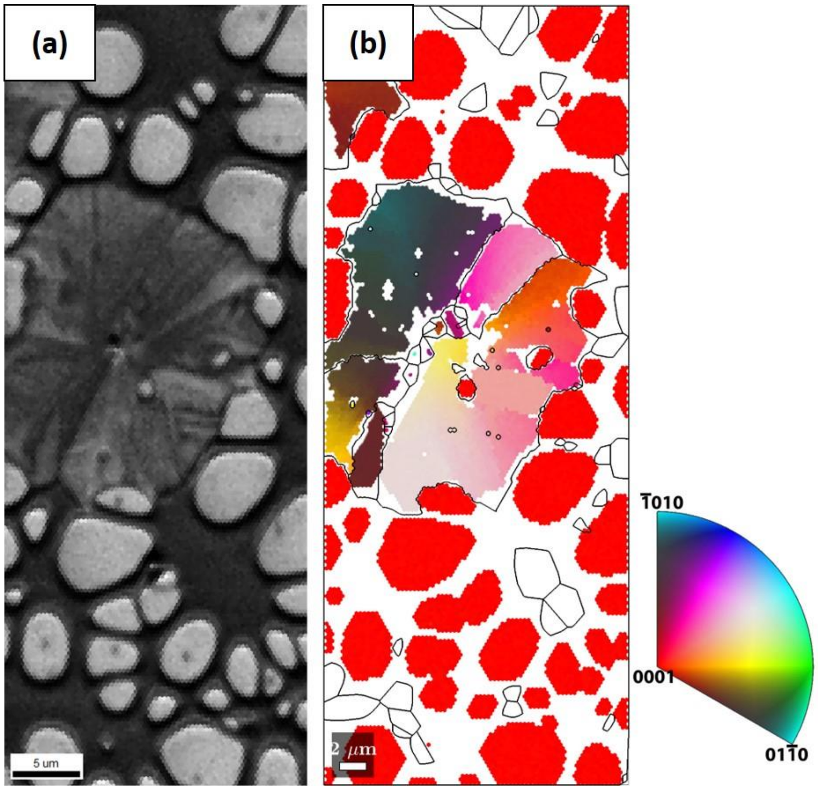

Fig. S 22. EBSD of crystalline $Si_{0.48}Ge_{0.52}O_2$ on STO substrate. (a) Image Quality map. They grey scale represents the degree of crystallinity. The STO is the brightest since it is single crystal. The darkest area is amorphous. And quartz crystals show intermediate contrast. (b) Inverse Pole Figure map of the same area as in (a) viewed along [001] direction. The color key is for the quartz crystal. Color gradient can be observed on the quartz crystal, suggesting the lattice of the crystal is rotating during the growth.

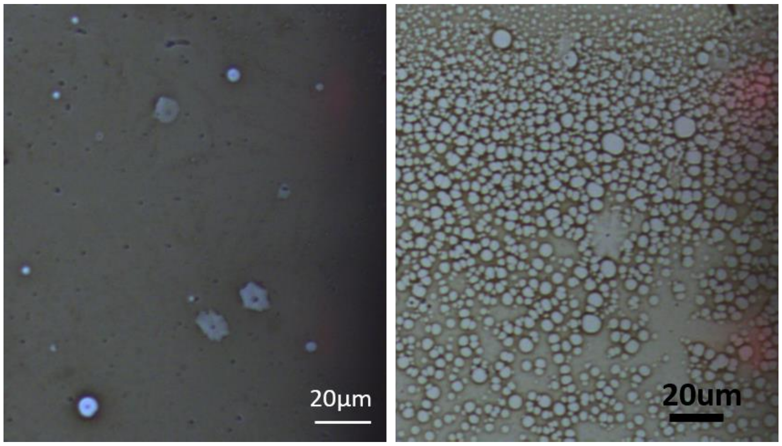

Fig. S 23. Optical microscopy images of thin films of $Si_{0.48}Ge_{0.52}O_2$ on STO substrates with (a) 3600 pulses and (b) 1800 pulses. It is clear that the amount of evaporation pits in the film with 1800 pulses are significantly higher.

Table S1. Compositions of the targets after synthesis, laser ablation, and the corresponding thin films.

Targets	Initial powders	After synthesis			After laser ablation			Thin films		
		Si%	Ge%	Cs%	Si%	Ge%	Cs%	Si%	Ge%	
(1)	GeO_2		100			100			100	GeO_2
(2)	SiO_2+GeO_2	31.11 $\pm$ 2.18	68.90 $\mp$ 1.49		30.27 $\pm$ 2.05	69.73 $\pm$ 1.46		7.89 $\pm$ 0.95	92.11 $\pm$ 4.28	$Si_{0.08}Ge_{0.92}O_2$
(3)	SiO_2+GeO_2	44.44 $\pm$ 2.73	55.56 $\pm$ 1.26		53.51 $\pm$ 3.16	46.50 $\pm$ 1.19		15.57 $\pm$ 1.43	84.43 $\pm$ 3.95	$Si_{0.16}Ge_{0.84}O_2$
(4)	SiO+GeO_2	70.60 $\pm$ 3.37	29.40 $\pm$ 4.85		73.59 $\pm$ 2.49	26.41 $\pm$ 1.33		75.37 $\pm$ 4.81	24.63 $\pm$ 1.50	$Si_{0.75}Ge_{0.25}O_2$
(5)	SiO_2+GeO_2	40.11 $\pm$ 2.60	58.41 $\pm$ 1.13	1.48 $\pm$ 0.43	43.84 $\pm$ 2.69	54.77 $\pm$ 1.04	1.39 $\pm$ 0.33	21.39 $\pm$ 2.12	78.61 $\pm$ 3.70	$Si_{0.21}Ge_{0.79}O_2$
(6)	SiO_2+GeO_2	45.38 $\pm$ 3.05	53.87 $\pm$ 1.17	0.74 $\pm$ 0.39	56.69 $\pm$ 3.31	42.43 $\pm$ 1.01	0.88 $\pm$ 0.38	19.81 $\pm$ 2.07	80.19 $\pm$ 3.80	$Si_{0.20}Ge_{0.80}O_2$
(7)	SiO_2+GeO_2	65.89 $\pm$ 3.57	32.80 $\pm$ 0.80	1.31 $\pm$ 0.38	80.12 $\pm$ 3.78	18.82 $\pm$ 0.63	1.06 $\pm$ 0.40	47.98 $\pm$ 3.69	52.02 $\pm$ 2.60	$Si_{0.48}Ge_{0.52}O_2$
(8)	SiO	100			100			100		SiO_2

Table S2. Evolitoin of the c lattice parameter of GeO_2 thin films on Z-cut quartz substrates with temperature.

Temperature(℃)	26	300	400	500	600	700	800
c(Å)	5.673 ± 0.021	5.674 ± 0.020	5.675 ± 0.017	5.670 ± 0.021	5.657 ± 0.024	5.657 ± 0.021	5.654 ± 0.023

Table S3. Lattice parameters of the $Si_xGe_{1-x}O_2$ films grown on Y-cut substrates.

x	0	0.08	0.16	0.20	0.21	0.48	0.75
$d_{10\bar{1}0}$(Å)	4.331 ± 0.007	4.326 ± 0.005	4.323 ± 0.003	4.321 ± 0.008	4.325 ± 0.006	4.306 ± 0.008	4.292 ± 0.005
c (Å)	5.618 ± 0.075	5.597 ± 0.032	5.572 ± 0.015	5.538 ± 0.023	5.547 ± 0.025	5.468 ± 0.023	5.412 ± 0.011
a (Å)	4.978 ± 0.065	4.976 ± 0.034	4.971 ± 0.014	4.949 ± 0.026	4.944 ± 0.028	4.917 ± 0.021	4.916 ± 0.009
